# Using consentless practices on medical wards—A qualitative study

**DOI:** 10.1177/09697330261459829

**Published:** 2026-06-29

**Authors:** Joar Björk, Niklas Juth, Tove Godskesen

**Affiliations:** 1 Centre for Research Ethics & Bioethics (CRB), Uppsala University, Uppsala, Sweden

**Keywords:** coercion, nursing, hospital staff, professionalism, consent, decision-making capacity

## Abstract

**Background:**

In contrast to legally mandated coercive care in psychiatry, there is a paucity of studies on the use of coercive practices within somatic healthcare.

**Aim:**

To gain an in-depth understanding of the practices used by nurses and other staff to provide somatic healthcare interventions to ill patients in the absence of informed consent.

**Research design:**

Focus-group interviews were held, and the collected data were analyzed qualitatively using Reflexive Thematic Analysis.

**Participants and research context:**

Participants (*n* = 37, mainly nurses) from medical wards at two Swedish hospitals.

**Ethical considerations:**

All participants were informed about the study’s aims and voluntary nature, and provided oral consent before being interviewed. No sensitive personal information was recorded.

**Findings:**

The qualitative analysis resulted in the identification of four main themes: *Consentless practices are used all the time*; *“Good examples” of consentless practices are learned on the ward*; *Choosing and adopting consentless practices to suit the situation*; and *Professional experience influences the use of consentless practices*.

**Conclusions:**

Consentless practices are frequently used on medical wards, and nurses and other staff are familiar with a wide array of such practices. Consentless practices are learned informally, mainly through observation. Staff with greater professional experience can deliver healthcare interventions without overt coercion, but may become habituated to using consentless practices. These findings raise a range of ethical concerns. Better opportunities for reflection and learning on this topic may improve the ethical climate on medical wards.

## Introduction

Respecting patient autonomy is a core ethical value in nursing and is consistently emphasized in the general bioethics literature.^1–3^ Registered nurses (hereafter: nurses) are expected to invite patients to participate in decision-making and to respect their right to refuse unwanted interventions.^4^ Healthcare staff (hereafter staff) should support patients in understanding their medical situations, refrain from controlling their decisions, and make sure that all interventions are preceded by informed consent.^5,6^

## Background

Despite these ethical ideals, real-world clinical practice does not always align with the ideals of autonomy.^7^ One of the most important practical implications of respecting patients’ autonomy is to insist that healthcare interventions should be preceded by informed consent. However, some patients’ current situations or underlying health conditions may limit their capacity to understand medical information, and others may be unwilling or unable to partake in shared decision-making.^8–10^ In situations where valid (present) consent is unavailable, staff may rely on patients’ previous preferences or substituted judgment. However, this approach is considered ethically inferior to present consent, and ethical dilemmas arise when patients are forced to accept interventions contrary to their current expressed preferences to honor previous preferences, substituted judgment, or perceived best interests.^11,12^ Despite the ethical challenges associated with providing healthcare interventions without valid consent, it would be ethically disastrous to withhold necessary treatment altogether. Patients who cannot provide valid consent may have serious medical needs, and individuals in this vulnerable state must not be left without attention.^13,14^ To address this issue, many countries have enacted legislation permitting some form of legally mandated coercive care. In Sweden, such legally mandated coercive care is primarily reserved for *psychiatric* care.^15^ Hence, providing *somatic* treatment without patients’ valid consent is generally illegal in Sweden, and no legal provision exists for others to issue consent on patients’ behalf.^16^

Nursing studies indicate, however, that patients in somatic healthcare settings in Sweden and elsewhere receive a wide range of interventions without valid consent, often with the involvement of nurses.^1,17,18^ Despite the fact that such consentless practices are a regular feature of clinical practice, studies suggest that staff seldom discuss this openly and often feel unsure about the ethical and legal context.^19,20^ Few studies have explored how staff in somatic healthcare, particularly nurses, navigate these situations or what strategies they employ to provide somatic healthcare interventions to ill patients when informed consent cannot be obtained. One prior article from the same research project, which instead looked at nurses’ ethical reasoning regarding consentless practices they have witnessed and/or participated in, has already been published.^21^

## Aim

The aim of this study is to gain an in-depth understanding of the practices used by nurses and other staff to provide somatic healthcare interventions to ill patients in the absence of informed consent.

## Methods

*Design:* The study had a qualitative, inductive approach, and the data were collected via focus group interviews.^22^

*Setting:* Participants were recruited from one small and one large somatic hospital in Sweden, both offering around-the-clock services. Medical wards were chosen for their varied patient demographics, particularly their large population of elderly patients with complex healthcare needs. Legally mandated coercive care is rare in this study setting.^23^

*Recruitment and participants:* Information about the study was posted on the ward, and participants were recruited through ward operation managers. Eligibility required a minimum of 1 year of clinical experience. Although nurses were the main participant group, other staff were invited to enrich the discussion with a variety of perspectives (Table 1). Henceforth, the term “participants” is used for participants in this study, and “staff” for healthcare staff in general.

**Table 1.** Participant demographics.

Number of focus group interviews	8
Total number of participants (participants per interview)	37 (4–6)
Professions represented (professional title abbreviation), number of participants	Registered nurses (RN), 13
	Assistant nurses (AN), 15
	Medical doctors (MD), 5
	Physiotherapists (PT), 2
	Occupational therapists (OT), 2
Sex distribution (female/male)	33/4
Age, mean (range)	38 (22–64)
Years worked on a medical ward, mean (range)	12 (1–48)

*Data collection:* Focus group interviews were conducted by the first author. A semi-structured interview guide was used (Table 2), which was developed based on insights from three pilot interviews with nurses on medical wards. The preliminary interviews indicated that the term “coercion” (Swedish: “tvång”) tended to inhibit open discussions, as nurses primarily associated it with legally mandated coercive measures in psychiatric care, something rarely encountered in their workplaces. Instead, the more encompassing term “consentless practices” was used as an interview prompt (Table 2). The researchers chose not to define this term precisely, nor to enforce any particular terminology. Open-ended questions were used, and participants were encouraged to develop their answers by follow-up questions.^22^ Audio recordings were made and were transcribed by an academic transcription company.

**Table 2.** Interview guide.

In this interview the term “consentless practices” will be used very broadly for actions employed to make patients comply with treatment. Examples include a broad range of actions such as coaxing, deception, non-voluntary medication and the use of physical force.
Are consentless practices, in this sense, used on this ward?
What kind of patients may be subject to consentless practices?
Can you describe some frequently used practices?
How do staff learn to use and master these practices?
What are your reflections about the usage of consentless practices?
Are situations involving consentless practices discussed among staff or by team leaders?

*Data analysis:* Reflexive thematic analysis, as described by Braun and Clark and developed by Byrne, was used as it facilitates the in-depth exploration of participants’ experiences and the understanding of complex phenomena.^24–26^

## Ethical considerations

Ethical approval for the study was requested from the Swedish Ethical Review Authority (registration no. 2022-00,142-01), which concluded that formal approval was not required. Individuals invited to participate received both oral and written information outlining the study’s purpose and the voluntary nature of their involvement. No sensitive personal data were collected. Prior to the focus group interviews, participants provided oral consent for participation as well as for the dissemination of the results. To ensure confidentiality, all personal identifiers were removed, and the findings are presented in a way that prevents the identification of individuals. Interview transcripts and the code key were stored separately from the audio recordings on two secure computers.

## Results

The results of the thematic analysis were synthesized into four main themes: *Consentless practices are used all the time; “Good examples” of consentless practices are learned on the ward; Choosing and adopting consentless practices to suit the situation*; and *Professional experience influences the usage of consentless practices*.

### Consentless practices are used all the time

Participants stated that they and their colleagues use consentless practices to provide healthcare interventions to patients who cannot consent to medical recommendations “all the time” or “every day.” Consentless practices were described as being frequently used on patients who have impaired decision-making capacity, and sometimes on fully competent patients. For instance, patients deemed competent but unwilling are sometimes compelled to participate in mobilization exercises or undergo urinary catheter placement. While many accounts focused on discrete actions (e.g., administering medication, assisting with hygiene), some participants also described care being administered for extended periods without the patient’s consent:

When they come [to the hospital] we do too much even though they’re like: “Why did you take me here? I was clear that I did not want to go to the hospital” – and still staff at the nursing home send them in, even though it is in their [care plan] that they do not want to go to the hospital (AN, interview C)

### “Good examples” of consentless practices are learned on the ward

Participants emphasized that consentless practices are not learned during professional training. Indeed, participants complained that this left them ill-equipped for working with patients on a medical ward. The learning of consentless practices occurs via peer-to-peer observation in which “good examples”—that is, “good” in the sense of “successful in making patients comply with treatment”—are noticed and added to one’s repertoire. Staff sometimes make recommendations to colleagues about what “works” on specific patients. These practices typically lack established terminology, and they are rarely discussed overtly. Their perceived effectiveness was context-dependent: what “works” with one patient may be ineffective with another. Mastery of such practices was understood as knowing which practices to use and when in order to deliver healthcare with minimum coercion. Learning new practices and diversifying one’s repertoire was seen as a form of professional development:

You have good examples that you have collected over the years. Ways that you’ve seen older colleagues effectively persuade patients. Well, you save these for the future (MD, interview D)

### Choosing and adopting consentless practices to suit the situation

Participants stressed that consentless practices are flexible tools that staff apply to suit each situation. Staff also frequently try out and shift between several different practices during one care interaction, to see what might be most successful. If patients do not comply with medical recommendations, staff commonly start by trying to motivate them. Terms participants used to describe this included “cheering on,” “encouraging,” “pushing,” “cajoling,” “coaxing,” and “psyching up.” Most ways of motivating were seen as acceptable, and although participants indicated that motivating could sometimes be problematic, they found it hard to explain when or why. Some practices, however, were described as deceptive and manipulative. For instance, staff may exaggerate the risks of non-compliance and overstate interventions’ importance to make patients comply. The range of alternatives open to patients is sometimes deliberately misconstrued:

First RN: You can keep them [patients] up and not just put them back into bed when they want to…

Second RN: We can say, like: “there is no colleague who can help me put you back into bed right now…” [facial expression shows this is not true]

First RN: (laughing, fills in) “…so you’ll just have to sit up a bit longer” (Two RNs, interview B)

Participants frequently described engaging in bargaining practices such as allowing patients to decide one thing, like when to shower, in return for their compliance with medical recommendations. Staff also use appeals to authority, including their own authority, “tradition,” and other staff, to make patients comply:

You can lie to the patient, saying that the doctor said that a particular thing needs to be done (RN, interview E)

Then I just said [in stern voice]: “This is the way it must be.” And the patient fell into line and could be admitted… so yes I use my physician’s authority when I really want them to accept my suggestions” (MD, interview D)

Another reported tactic involved using relational bonds between patients and members of staff, or between patients and their relatives, to increase patients’ compliance. Having a good relationship with patients was stressed as an important instrument to make patients comply. If a patient refuses recommendations, staff may ask someone else to step in, who may have a greater chance of making the patient comply. Participants also noted that staff may use relational tactics, such as flirting with a patient, to make them accept.

Additionally, many practical measures are used to deliver healthcare in the absence of consent. These include hiding medication in food or using distracting techniques to obtain blood samples. Some tests, such as cognitive assessments, can be undertaken even if the patient refused to be tested, by replacing formal testing with observation of the patient’s behavior. Participants described working in pairs to use distraction tactics, with one person capturing the patient’s attention while another performs the intervention. Another measure described was nudging the patient step-by-step rather than asking them upfront about something they might object to:

Like you agree they don’t need to shower, only use deodorant. But then, when they sit at the toilet, you take the shower handle and say “can you hold this for a second”. And you coax a little. So all of a sudden they are showering. Just because I did that “no shower, only deodorant” thing… (AN, interview C)

Participants frequently described situations in which they did not ask for patient consent. They stated that staff often make medical decisions which are subsequently communicated to patients, rather than involving patients in the decision-making process. Physicians often make these decisions at a time and place removed from their actual administration and either inform patients briefly or leave informing them to the nurses. Similarly, quick medical interventions such as drawing blood or checking blood pressure can be carried out without any questions being asked. Participants emphasized that staff unlearn asking the patient as they learn to do the day’s tasks faster:

To get it the way that we [staff] want, which is also good for the patient, we say “here is what we’ll do” instead of asking “do you want this?” That often works better (RN, interview E)

Being attentive to timing and situational cues was described as a way to increase patients’ compliance with staff’s intentions. Participants expressed that many patients respond differently to the same proposal on different occasions due to contextual features such as mood, alertness, or who made the proposal. Such variation can be exploited by staff. Participants stressed that a patient’s “no” should not be interpreted as their true, final, or enduring decision. On the contrary, it may often pay off to repeat the same suggestion:

You simply approached the patient at the wrong time, and nothing [no intervention] would have been acceptable because the patient was sad or something. So they say no to everything. Then you just have to come back later to try again (OT, interview H)

Persistent prompting or “nagging” was described as a commonly used measure to make patients cooperate. Just as with motivation, participants expressed that it is hard to know whether patients ultimately accept interventions because they have changed their minds, or simply to stop the nagging.

Sometimes patients are also made to comply through physical force, for instance by putting them in a wheelchair and pushing them to another location, by holding patient’s hands still or holding the patient down while inserting peripheral vascular catheters, urinary catheters or nasogastric tubes. Using physical force was seen as less attractive to all parties:

It is difficult for us, but you remind yourself … it is for their own good. It is dangerous to have so much urine in the bladder. Yes, it is difficult to do it, I mean forcing somebody. I am a woman too. I too… I too would dislike it [being done to me] (AN, interview E)

Participants explained that staff hone their intuition over time regarding which measure or combination of practices to apply in a given situation, and that individual staff members tend to develop different repertoires. Staff who work together for some time know which practices their colleagues use. Staff who use highly coercive practices are seen as less skilled and/or less ethical. At the same time, the participants supported the right of staff to set their own limits on what they will and will not do.

### Professional experience influences the usage of consentless practices

According to participants, the extent of a staff member’s professional experience influences how they apply consentless practices. Experience seems to influence consentless practices in complex and seemingly contradictory ways. Staff with more professional experience can adapt their working methods to different patients to avoid using harsher forms of consentless practices. However, staff with more experience were also said to grow accustomed to providing healthcare without patients’ consent:

Older [staff] who have worked longer have more experience and are used to doing the same thing all the time, so they kind of do it without thinking so much [about patients’ consent] (…) Sometimes it goes a bit too fast, like they just follow their own heads (AN, interview G)

In contrast, staff with less professional experience were portrayed as being more attuned to patients’ expressed wishes. Participants described recently trained staff as more focused on patients’ self-determination. The modern, consent-oriented mode of working was seen as more respectful. At the same time, participants noted that this may be time-consuming:

Those of us who have not been working as long take more time, perhaps we pamper patients too much (…) Like you wait for the patient, and you ask them what they want. You explain why you are there and why you want to do the things you want to do (RN, interview G)

## Discussion

Participants generally showed extensive experience with using consentless practices to make patients comply with treatment. These practices were described as part of the everyday routine on medical wards, and participants explained that staff learn and become accustomed to a broad range of consentless practices through informal learning on the ward.

This study corroborates earlier indications that consentless practices commonly occur even outside of legally mandated (psychiatric) coercive care.^16,27^ The practices described here align well with findings from other countries and contexts (for an overview, see^28^), which is notable as participants emphasized that these strategies are not learned from textbooks but acquired informally. This presents an apparent paradox: although these strategies are locally developed, often as pragmatic responses to encourage patients’ compliance in a specific care setting, they appear remarkably consistent across diverse contexts. Some practices described in this study appear to be novel, however, like the conscious avoidance of asking for patients’ preferences as a strategy for providing healthcare without consent. Although the failure to elicit preferences is well discussed in the nursing literature,^29^ it is rarely framed as a means of making patients comply. This strategy is at odds with the principles of “shared decision-making,”^30–32^ which stress patients’ comprehensive involvement throughout the decision-making process, in contrast to simply providing patients the opportunity to accept or reject ready-made options.^33,34^ Also, there is a risk that nurses and other staff get insufficient insight into the patients’ values, preferences, and reasoning. This lack of understanding may compromise their ability to assess whether the patient possesses decision-making capacity, and whether the intervention aligns with the patient’s wishes.^35^ Indeed, if nurses provide consentless care in part because they are afraid that patients may not be adherent to treatment recommendations, failure to elicit preferences seems to miss the goal as it means nurses will not know whether the patient would have been adherent, if given an opportunity.

Furthermore, avoidance of asking represents a deviation from core nursing ideals. Our results indicate that nurses, at times, abandon a stance grounded in interaction and attentive listening in favor of one of commanding and controlling, simply because it is perceived to “work better” (in one participant’s words). This is in stark contrast to the ideal of enabling patients to participate in their care.^36^ Indeed, proximity, not distancing, should be a nurse’s ideal.^37^ There is also a risk that the described attitude hampers relationship-building between nurses and patients,^38^ which is, arguably, more important in situations where there is a risk of consentless practices.^39^ We also want to emphasize that although seeking informed consent is a very important aspect of autonomy, consent by no means equates or exhausts the demands placed on nurses by respect for autonomy.^40^ Indeed, establishing *rapport* with the patient and being respectfully present are also vital to high-quality and autonomy-respecting nursing.^41^ Indeed, the indication that some nurses feel that not asking for patients’ preferences “works better” signals that consentless care is perhaps not driven by a fear that patients may not adhere to recommendations, but rather that nurses feel so pressured for time that perceived “slow” tasks have to be avoided. This is an alarming sign that the quest for efficiency may become counter-productive as well as threatening the human qualities in care that nursing stands for.^42–45^

Another novel finding of this study is that alongside the administering of specific interventions without consent, participants relayed stories of whole hospitalizations occurring without consent, even among patients with intact decision-making capacity. Indeed, elderly patients seem to be at risk of consentless interventions regardless of their decision-making capacity. This may be due to low expectations of elder patients’ decision making capacity, or older patients’ difficulty to assert their rights. In any case, this potential ageism is in stark contrast with nursing ideals, and with respecting the human rights of *all* patients regardless of age.^46–48^

A clear narrative emerging from this study is that nurses and other staff consider the mastery of practices for making patients comply with treatment as a matter of professional competence and creativity. This view can be recognized in previous research.^49,50^ Participants also asserted that this competence is not learned during university training, which may tie into the discussion of why recently graduated nurses feel ill-equipped for their work,^51^ and help to explain why more experienced staff use consentless practices differently. That staff may become increasingly tolerant towards consentless practices by habituation has been indicated by some studies,^52,53^ while other studies suggest a more complex dynamic.^54,55^ If one wishes to take a positive view, this may indicate that nurses are not destined to become disinhibited to consentless practices.

The way participants discussed learning and using consentless practices invites comparison to *tacit knowledge* and *phronesis* as models of professional competence.^56–58^ In line with Polanyi’s observation that “we can know more than we can tell,”^58^ ^pg 4^ participants expressed a wealth of experience of consentless practices but also that such practices remain largely tacit—unnamed, rarely discussed, and neither formally taught nor systematically learned. Tacit knowledge overlaps with experience-based intuitive knowledge, as discussed by Patricia Benner,^59^ and staff seem to use intuition to choose among many potential strategies in a given situation. Indeed, participants could not articulate any rules for when to use different practices. The term “phronesis” comes from Aristotle’s analysis of knowledge, and denotes the capacity to judge which practices need to be taken in uncertain situations where simple rules do not apply.^60^ The array of consentless practices functions as a flexible tool kit for dealing with various challenges that arise when consent is lacking, and practices are learned by observing role models; both of these features align with writing on phronetic knowledge.^61^ So, too, does participants’ contextualized discussion of the efficiency of different practices, which were not understood as (in-)efficient per se but as more or less fitting to a situation. At the same time, phronesis is, by definition, practical wisdom tending to lead to morally defensible actions.^62^ This does not seem to be true of all actions described in this study. By and large, then, the use of consentless practices may *function* as a phronetic competence but with no guarantee of ethical quality. One may speculate whether increased individual and collective self-reflection could be of help as reflection is generally considered necessary for the development of phronesis^63^ and for good nursing ethics in general yet seems scarce in this context.

The authors of this study—all of whom are ethicists—do not claim that using consentless practices in somatic care is always unethical. As many patients lack the capacity to consent but have a clear need for medical treatment, consentless practices cannot be avoided altogether.^64^ However, we are alarmed by indications that even patients with intact decision-making capacity may be subjected to consentless practices,^17,65^ and that the usage of such interventions appears, in this context, to be largely left to nurses’ (and others’) individual discretion. Also alarming is the suggestion that the nurse-patient relationship, a crucially important element in nursing, is at times used as a tool to manipulate patients. While it may not be entirely possible to avoid such dynamics, we believe that greater ethical reflection on the implications and potential consequences is warranted.

We would like to conclude with a bold suggestion. If consentless practices cannot be entirely avoided in somatic healthcare, then perhaps staff should be systematically trained to use—in such situations—those practices that are least ethically abrasive. Previous studies suggest that staff would welcome support in navigating these ethically complex situations.^13,20,49^ Participants in this study indicated that there is an ethical difference between mild motivation practices such as “encouraging,” and, for instance, insistent nagging. Awareness of such distinctions offers a possible entryway for ethical analysis and training. If nurses and others learn the compassionate use of mild consentless practices, when necessary, this may reduce the usage of more oppressive measures. At the same time, we underscore that all attempts to engage staff in discussion or training on this topic must acknowledge the complex and somewhat contradictory understanding of consentless practices evidenced in this study, whereby staff partly discuss such practices in the ethical sense (i.e., good/bad) and partly in a pragmatic sense (i.e., efficient/inefficient). When we speak of training it must be avoided that staff focus on the latter aspect, as this could lead to ethically disastrous results. To state the obvious: we do not advocate training staff to become yet more efficient at providing healthcare without consent. Nurses and other staff already have tremendous power over patients, and such power advantages must be used in a responsible way.

## Methodological considerations

This study’s sample included staff representing various professions, and the use of focus group methodology facilitated rich, diverse and interactive discussions. However, the focus group format may also have limited the expression of sensitive or dissenting views, particularly in the presence of professional hierarchies. Participants were recruited through the ward operation managers, which may have resulted in self-selection of individuals with a particular interest in ethical issues. Researcher triangulation was employed during the data analysis to enhance the depth and credibility of the findings. JB is a physician and clinical ethicist, NJ is an academic ethicist, and TG is a registered nurse and clinical ethicist. The research team brought complementary perspectives to the data analysis to obtain a rich perspective. The introduction of the term “consentless practices” may be considered both a strength and a limitation. During the pilot testing of the interview guide, the term “coercion” blocked rather than encouraged discussions among staff. When this term was replaced by the broader notion “consentless practices,” participants were more forthcoming in their discussions. As evidenced by the discussion, the (perceived) lack of words to talk about consentless practices was keenly felt by participants, and the researchers’ efforts to avoid fixed definitions seemed to make discussions easier. Indeed, participants were able to discuss a wide range of relevant practices. An upshot of speaking of “consentless practices” was that discussions were not limited to consent procedures and judgments of (in)capacity, but also included practices which bypassed such issues altogether. At the same time, this analytical choice and the lack of standardized terminology may make comparison with previous studies more difficult. Further, the fact that the interviewer is a clinical ethicist may have made some participants reluctant to share more problematic practices. As the study was conducted in medical wards at two hospitals, the transferability of the findings to other institutional or cultural contexts should be approached with caution.

## Conclusions

In this qualitative study exploring nurses’ and other staff’s experience of providing healthcare without patients’ consent, participants reported that this is part of everyday practice on medical wards. They displayed extensive familiarity with a wide array of consentless practices and explained how these are learned informally at the ward. Being able to tailor the usage of such practices to a wide set of clinical situations was seen as an important professional competence. More experienced staff may have greater skill at delivering care without overt coercion, but may also be less inhibited about employing consentless practices. In some cases, whole hospitalizations are provided without consent, even to patients with intact decision-making capacity. Among other practices, participants described how the avoidance of asking can be used to make patients comply with interventions. Further studies using quantitative study designs could investigate whether, for instance, there are differences between professional groups when it comes to usage of practices. The results were analyzed in light of scholarly writing on *phronesis* as a form of professional competence. The possibility of training staff in the judicious use of consentless practices was discussed.

## Appendix

### Abbreviations

RN: Registered nurse
AN: Assistant nurse
MD: Medical doctor
PT: Physiotherapist
OT: Occupational therapist
